# High-Fat Programming of Hyperglycemia, Hyperinsulinemia, Insulin Resistance, Hyperleptinemia, and Altered Islet Architecture in 3-Month-Old Wistar Rats

**DOI:** 10.5402/2012/627270

**Published:** 2012-09-05

**Authors:** Marlon E. Cerf, Charna S. Chapman, Johan Louw

**Affiliations:** Diabetes Discovery Platform, South African Medical Research Council, P.O. Box 7505, Tygerberg, Cape Town 7505, South Africa

## Abstract

High-fat programming, by exposure to a high-saturated-fat diet in utero and/or during lactation, compromises beta-cell development and function in neonatal and weanling offspring. Therefore, high-fat programming effects were investigated on metabolism and islet architecture in young adult rats. Three-month-old male and female Wistar rat offspring were studied: HFG (maintained on a high-fat diet throughout fetal life), HFP (high-fat diet maintenance from birth to 3 months), and HFGP (high-fat diet maintenance throughout fetal and postnatal life). Control rats were maintained on a standard laboratory diet. Pancreata were double immunolabeled for insulin and glucagon to assess islet morphology and with Ki-67 to determine islet and acinar cell proliferation. HFP and HFGP males were heavier, hyperleptinemic, and hyperinsulinemic. Hyperglycemia presented in HFP males, HFP females, and HFGP males. HFGP males and HFP females were insulin resistant. HFP males displayed beta- and alpha-cell hyperplasia with alpha-cell hypertrophy evident in HFP females. Acinar cell proliferation rates were increased in HFP males. Postnatal high-fat programming induced the most diabetogenic phenotype with high-fat maintenance throughout fetal and postnatal life resulting in a severely obese phenotype. Fetal and postnatal nutrition shapes offspring health outcomes.

## 1. Introduction

The pancreatic beta-cells secrete insulin in response to fluctuations in circulating glucose concentrations to stimulate glucose uptake into peripheral organs. Beta-cell failure (insufficient insulin secretion) and insulin resistance (impaired action of insulin to stimulate glucose uptake) precede the development of type 2 diabetes. Both beta-cell failure and insulin resistance contribute to hyperglycemia, the hallmark of type 2 diabetes. Alpha-cells secrete glucagon which is antagonistic to insulin and help to regulate glucose homeostasis.

Obesity is strongly associated with insulin resistance. Leptin, an anorexic hormone, secreted by adipose tissue, binds to the appropriate leptin receptor triggering intracellular signaling processes in the ventromedial hypothalamus (satiety center) which in turn provides a negative feedback system to inhibit caloric intake and prevent obesity [1, 2]. In obese states, leptin is oversecreted resulting in hyperleptinemia whereby leptin does not elicit its anorexic effects. 

Environmental factors during intrauterine development may alter the structure, physiology, and metabolism of the body [3]. Developmental programming can be induced by manipulating maternal nutrition and investigating the effects in offspring. Altered maternal/fetal metabolism appears to be associated with a diabetogenic effect in adult offspring resulting in permanent deficiency of endocrine pancreatic function [4]. Rat offspring maintained on a maternal high-fat diet were hyperglycemic and hyperinsulinemic [5, 6] with elevated blood triglyceride concentrations, increased body fat, increased hepatic weight, and triglyceride content [7] and exhibited vascular dysfunction [8]. Insulin resistance in offspring is consistently programmed by maternal dietary imbalance [9]. High-fat programming, by exposure to a high-saturated-fat diet in utero and/or during lactation, compromised beta-cell development, and function in neonatal [10, 11] and weanling rats [12–14]. This study therefore sought to determine the effects of maintenance on a high-fat diet in utero and/or during postnatal life on body weight, fasting circulating leptin, glucose and insulin concentrations, HOMA, and on beta- and alpha-cell number, size, and volume in 3-month-old (young) adult rats. Cell proliferation occurs to replenish cell populations therefore islet cell and acinar cell proliferation were also assessed. The physiology and pathophysiology differ between males and females beyond reproductive function to include all physiological systems [15]. Therefore, programming effects in male and female offspring were investigated.

## 2. Materials and Methods

### 2.1. Experimental Design

Female Wistar rats were individually caged with free access to food and water at the South African Medical Research Council (MRC) Primate Unit (Cape Town, South Africa) in a room at a temperature of 24°C with a 12 : 12 h light-dark cycle. Upon mating with pregnancy confirmed with the presence of vaginal plugs, mothers (*n* = 3-4 per group) were fed either a low-fat-control (10% fat as energy) or high-fat (40% fat as energy) diet; offspring were therefore maintained on either a control or high-fat diet during defined periods of fetal and postnatal life. At 3 weeks of age, the offspring were weaned and directly fed either control or high-fat diets. Male offspring and female offspring were studied separately. The 3-month-old male and female offspring studied were: HFG (gestational high-fat diet), maintained on a high-fat diet throughout gestation (throughout fetal life only) and a control diet throughout lactation and postnatal life); HFP (postnatal high-fat diet), maintained on a control diet throughout gestation and a high-fat diet from birth to 3 months (throughout lactation and postnatal life); and HFGP (gestational and postnatal high-fat diet), maintained on a high-fat diet throughout gestation, lactation, and postnatal life). The control rats were maintained on a standard laboratory diet throughout fetal and postnatal life. The standard laboratory (control) diet comprised 10% fat, 15% protein, and 75% carbohydrate (2.6 kcal/g) whereas the high-fat diet contained 40% fat, 14% protein, and 46% carbohydrate (2.06 kcal/g). The experiments were approved by the Institutional Ethics Committee and rigorously adhered to the National Institutes of Health Ethical Guidelines for the care and use of laboratory animals.

### 2.2. Determination of Metabolic Parameters: Body Weight, Fasting Serum Leptin, Blood Glucose and Serum Insulin Concentrations, HOMA-Insulin Resistance, and HOMA-Beta-Cell Function

Offspring metabolic outcomes were assessed at 3 months. Body weight was measured on a laboratory scale. Before determination of the circulating metabolic parameters, the offspring were fasted for 16 h overnight. Fasting serum leptin (rat leptin RIA kit, Linco Research, St. Charles, MO, USA), blood glucose (glucometer, Precision QID, MediSense, Oxfordshire, UK), and serum insulin (rat insulin RIA kit, Linco Research) were measured. HOMA-insulin resistance ([fasting plasma glucose (mmol/L) × fasting serum insulin (mU/L)]/22.5)) and HOMA-beta-cell function ([20 × fasting insulin (mU/L)]/glucose (mmol/L) − 3.5)) were calculated.

### 2.3. Immunolabeling and Image Analysis

Whole pancreata were harvested for immunolabeling experiments. Serial sections, 4 *μ*m^2^ thick, were selected for immunolabeling. Slides were analyzed from at least one animal per litter. Alpha-cells were first immunolabeled with a polyclonal glucagon antibody (Dako, Carpinteria, CA, USA) followed by immunolabeling of the beta-cells with a monoclonal insulin antibody (Sigma ImmunoChemicals, St Louis, MO, USA) as previously described [10, 13].

For image analysis, a Canon Powershot S40 digital camera (Canon, Tochigi, Japan) mounted on an Olympus BX60 light microscope (Olympus, Tokyo, Japan) attached to a personal computer was used to capture images. The camera focus, light intensity, and image resolution were controlled remotely and the acquired images were transferred to the computer using Remote Capture Software from Canon. The final digitized images were all 768 × 1024 pixels. Using the ×10 objective, images were acquired for alternate fields of view until the entire section was covered. For each field of view, the islets were captured using a ×40 objective for nuclei counts. An islet was defined as a cluster of eight or more endocrine cells. Image analysis was performed with the Leica Qwin Plus Software (Leica, Cambridge, UK).

Tissue parameters were measured using either an interactive option or color segmentation. Total tissue area was determined by adding the tissue measured in each field of view, using the interactive measurement option of the Leica software. Total islet area, total beta-cell area, and total alpha-cell area were determined by color segmentation and thresholding. Beta-cell number was assessed by counting the number of beta-cell nuclei. Beta-cell size was calculated by dividing the measured beta-cell area by the number of beta-cell nuclei counted and expressed in *μ*m^2^. The relative beta-cell volume was obtained by calculating the ratio between the area obtained by immunoreactive beta-cells and the area occupied by total islet cells. The same procedures were applied to estimate the alpha-cell number, size, and volume. The ratio of beta-cell area to alpha-cell area (beta-cell : alpha-cell) was determined by dividing the total beta-cell area measured by the total alpha-cell area measured. The inverse calculations were applied to determine the alpha-cell : beta-cell ratio.

### 2.4. Islet and Acinar Cell Proliferation

Proliferation indices of islet and acinar cells were calculated after immunostaining with the proliferation marker, Ki-67 (MIB-5; DakoCytomation, Glostrup, Denmark). A minimum of 500 nuclei of each cell type (islet and acinar cells) were counted. The number of positive proliferative cells was divided by the total cell number of cells to determine the islet and acinar cell proliferation indices. Cell proliferation was not assessed in female offspring.

### 2.5. Statistical Analysis

One-way ANOVA and Bonferroni's posttest were applied to determine differences in male and La Jolla, female offspring (GraphPad Prism 5, GraphPad Software, CA, USA). Genders were analyzed separately. Data are reported as means ± SEM with significance established at *P* < 0.05.

## 3. Results

### 3.1. Male Offspring Phenotype

#### 3.1.1. Metabolic Parameters

HFP and HFGP males were heavier, hyperleptinemic, and hyperglycemic compared to control males (Table 1). Further, HFGP males were heavier and hyperleptinemic compared to HFG and HFP males and hyperinsulinemic and insulin resistant compared to control and HFG males (Table 1). 

#### 3.1.2. Islet Morphology

**Figure 1:**
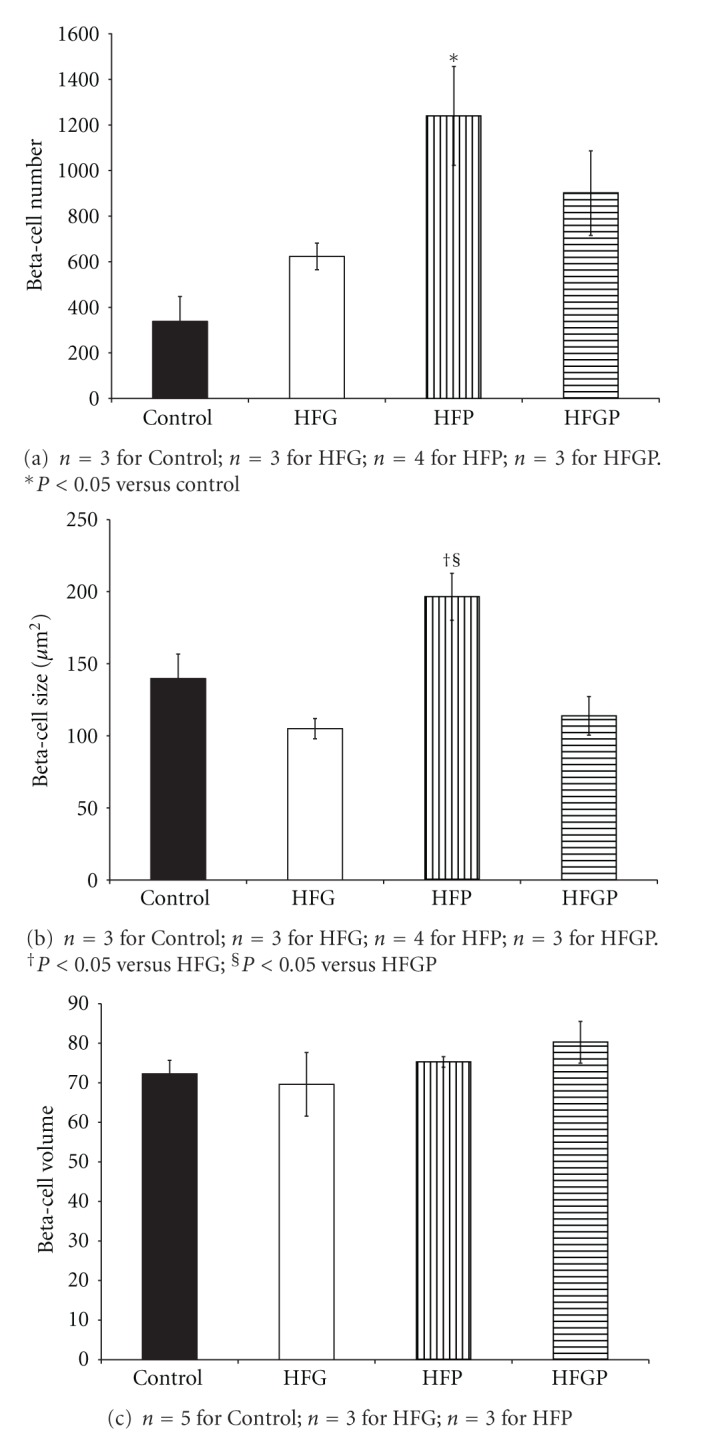
Beta-cell number (a), size (b), and volume (c) in 3-month-old male offspring maintained on a high-fat diet during fetal and/or postnatal life. HFG: high-fat diet during fetal life; HFP: high-fat diet during postnatal life; HFGP: high-fat diet during both fetal and postnatal life.

**Figure 2:**
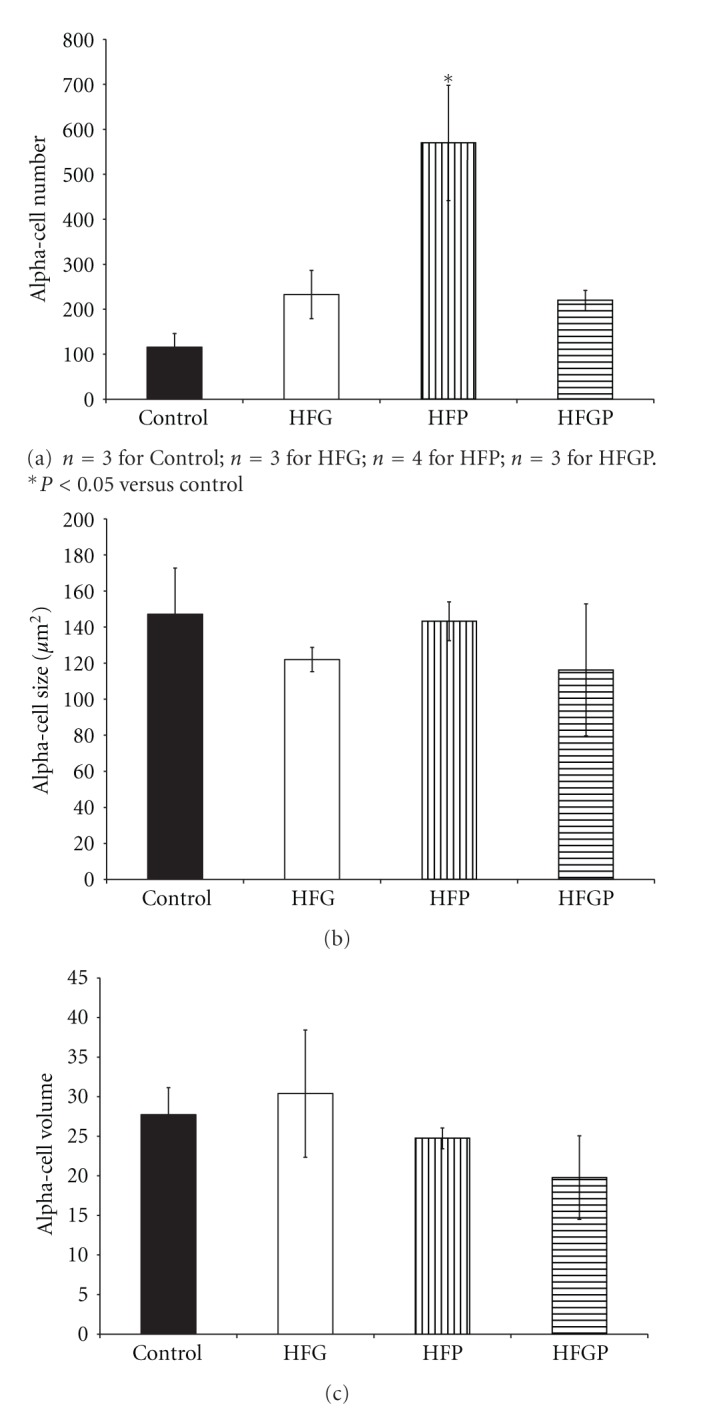
Alpha-cell number (a), size (b), and volume (c) in 3-month-old male offspring maintained on a high-fat diet during fetal and/or postnatal life. HFG: high-fat diet during fetal life; HFP: high-fat diet during postnatal life; HFGP: high-fat diet during both fetal and postnatal life.

In HFP males, beta-cell hyperplasia (Figure 1(a)) presented compared to control males with beta-cell hypertrophy (Figure 1(b)) relative to HFG and HFGP males. There were no changes in beta-cell volume in male offspring (Figure 1(c)). HFP males displayed alpha-cell hyperplasia (Figure 2(a)) compared to control males with no changes in alpha-cell size (Figure 2(b)) and volume (Figure 2(c)), beta-cell to alpha-cell ratio or alpha-cell to beta-cell ratio in male offspring (Table 2). There were no changes in islet cell proliferation; however, acinar cell proliferation was increased in HFP males compared to control, HFG, and HFGP males (Table 2). 

### 3.2. Female Offspring Phenotype

#### 3.2.1. Metabolic Parameters

Unfortunately, the female HFGP phenotype could not be characterized due to low sample numbers, therefore, comparisons were limited to control, HFG, and HFP females. Despite no significant changes in weight or insulinemia, HFP females were hyperleptinemic, hyperglycemic, and insulin resistant compared to control females (Table 1). Further, HFP females were also hyperglycemic and insulin resistant compared to HFG females (Table 1). 

#### 3.2.2. Islet Morphology

**Figure 3:**
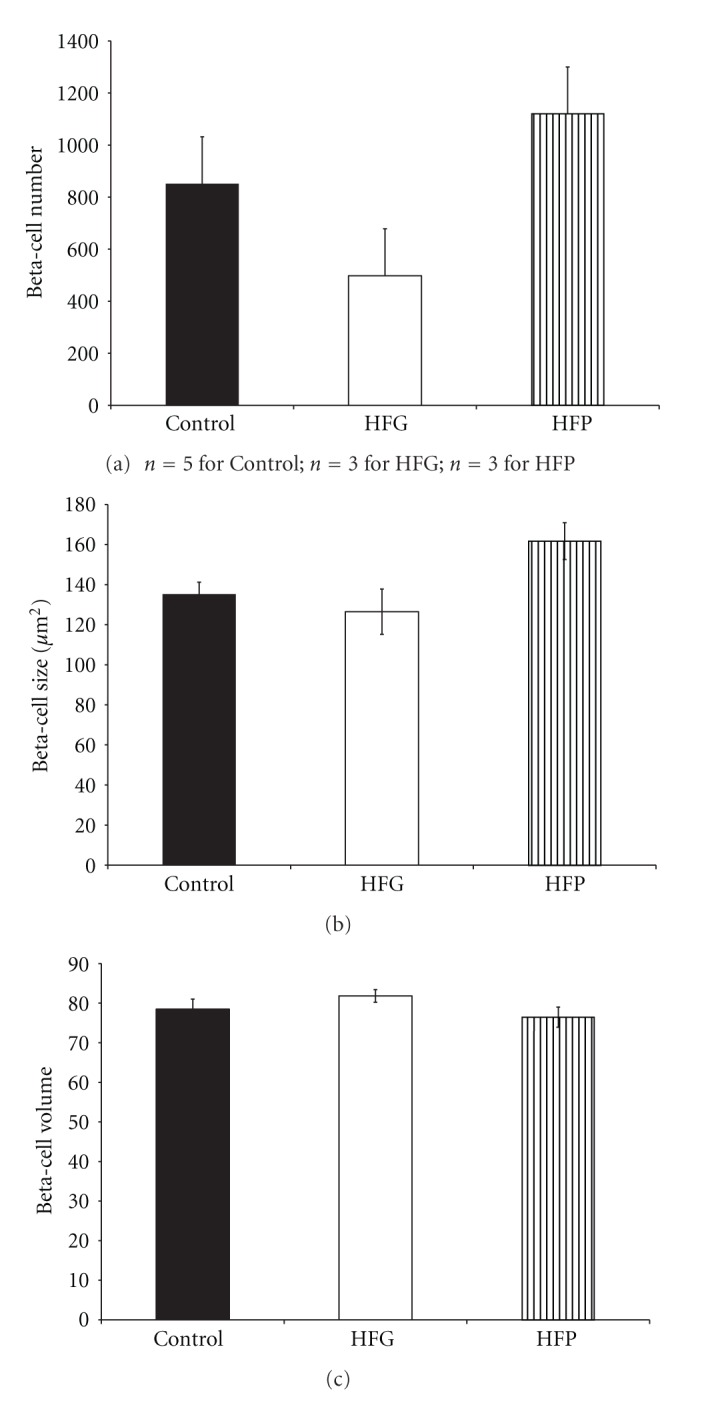
Beta-cell number (a), size (b), and volume (c) in 3-month-old female offspring maintained on a high-fat diet during fetal or postnatal life. HFG: high-fat diet during fetal life; HFP: high-fat diet during postnatal life.

**Figure 4:**
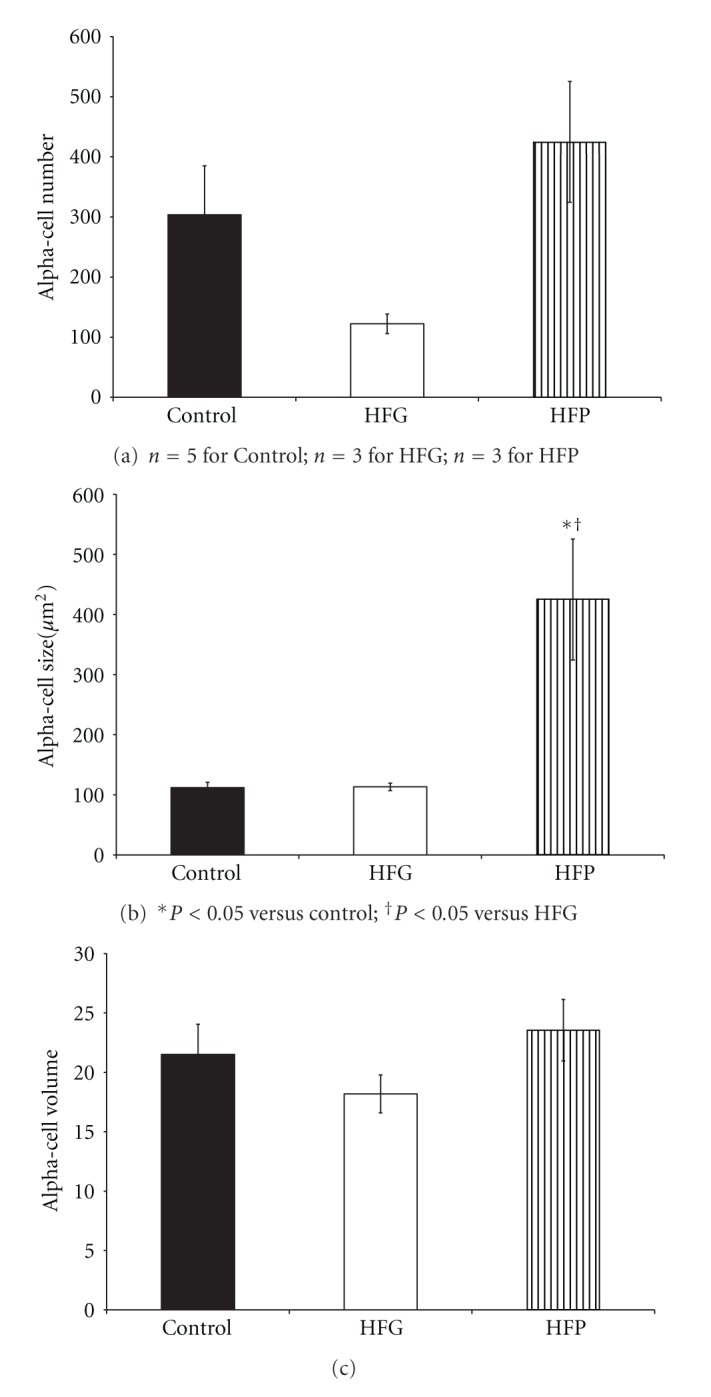
Alpha-cell number (a), size (b), and volume (c) in 3-month-old female offspring maintained on a high-fat diet during fetal or postnatal life. HFG: high-fat diet during fetal life; HFP: high-fat diet during postnatal life.

There were no changes in beta-cell number (Figure 3(a)), size (Figure 3(b)), or volume (Figure 3(c)) or in alpha-cell number (Figure 4(a)) in female offspring. However, HFP females presented alpha-cell hypertrophy compared to control and HFG females (Figure 4(b)). Further, there were no changes in alpha-cell volume (Figure 4(c)), beta-cell to alpha-cell ratio or alpha-cell to beta-cell ratio (Table 2). Islet cell and acinar cell proliferation rates were not determined in female offspring. 

## 4. Discussion

High-fat programming generated different phenotypes in young adult rats. Firstly, in male offspring, postnatal high-fat programming from birth to adulthood induced the most adverse effects: a diabetogenic phenotype characterized by hyperglycemia and a mild obese state with increased body weight and hyperleptinemia. Further, this diabetogenic state triggered an islet cell compensatory response evident by beta- and alpha-cell hyperplasia and increased acinar cell proliferation. In female offspring, postnatal high-fat programming induced hyperglycemia, hyperleptinemia, and alpha-cell hypertrophy. Secondly, high-fat maintenance throughout gestation and postnatal life resulted in a severe obese phenotype characterized by the highest body weights, leptin and insulin concentrations, and most severe insulin resistance in male offspring. Finally, gestational high-fat programming induced no changes in either metabolism or islet architecture presenting a normal phenotype with the absence of a diabetogenic and/or obese phenotype in both male and female offspring. 

In HFP male offspring, the beta- and alpha-cell hyperplasia reflects a compensatory increase in islet cell number likely in an attempt to restore normoglycemia. Glucagon, secreted by alpha-cells, is antagonistic to insulin and stimulates glucose release which potentially contributed to the hyperglycemia in HFP males. Beta-cell compensation is inadequate: beta-cell hyperplasia is countered by alpha-cell hyperplasia, and the absence of beta-cell hypertrophy results in no compensatory increase in beta-cell volume. Therefore, hyperglycemia persists in HFP males.

In HFP males, beta- and alpha-cell hyperplasia concomitant with an increase in acinar cell proliferation may mark acinar cells as a source for islet cells. The combination of the transcription factors, MafA, Pdx1, and Ngn3 was reported to drive the reprogramming of acinar cells to islet cells in the pancreas of adolescent immune-compromised mice [16]. Acinar and ductal cells coinhabit and largely populate the pancreas (islets only represent 1-2% of the pancreas). With similar lineages during ontogeny, the transdifferentiation of these nonislet pancreatic cells to beta-cells is attractive since they reside in close proximity to islet cells. 

Interestingly, HFP males demonstrated beta-cell hyperplasia relative to HFG and HFGP males which provided further evidence of beta-cell compensation. Compared to HFGP males, beta-cell hyperplasia in HFP males was independent of changes in glycemia and insulinemia suggesting that more beta-cells were required to maintain similar states of glucose homeostasis. With no significant differences in glycemia and insulinemia in HFG males, which were similar to the levels in control males, no islet compensatory response was necessary which may account, in part, for the beta-cell hyperplasia in HFP males relative to HFG males. 

Alpha-cell hypertrophy presented in HFP females in the absence of any changes in beta-cell morphology. It appears that postnatal high-fat programming therefore only minimally affects islet morphology in female offspring, but does increase alpha-cell sizes; this requires further investigation. 

A severely obese phenotype presented after high-fat maintenance throughout fetal and postnatal life generating the heaviest, most hyperleptinemic, hyperinsulinemic, and insulin-resistant male offspring that clearly demonstrated the adverse metabolic effects of long-term maintenance on a high-fat diet. Insulin and leptin are secreted into circulation in proportion to adipose tissue [17] indicative of obesity in HFGP males. An altered metabolic state of increased insulin demand, such as obesity which is strongly associated with insulin resistance, confers susceptibility to develop metabolic disease. The obese phenotype in HFGP males concurs with a study where hyperphagia in obese rats was associated with hyperinsulinemia and hyperleptinemia suggesting central resistance to insulin and leptin [18], and with another study where 12 weeks of high-fat feeding in mice was reported to induce both insulin and leptin resistance [19]. Beta-cells initially increase insulin secretion in response to insulin resistance [20] which may account for the hyperinsulinemia in HFGP males. Despite these adverse metabolic effects, islet morphology in HFGP males remained surprisingly intact. It was expected that the severe obese phenotype induced by high-fat maintenance throughout fetal and postnatal life would be associated with beta-cell hyperplasia and hypertrophy to compensate for their obese-insulin-resistant state. The current beta-cell population in HFGP males therefore appears to hyperfunction by increasing insulin secretion without restoring normoglycemia. Therefore, in HFGP males, islets appear to adapt to long-term high-fat maintenance despite their obese state. The islet cell morphology, although intact at this young adult life stage, is however predicted to deteriorate as the HFGP males age while still maintained on a high-fat diet.

A diabetogenic and/or obese phenotype was absent in HFG male and female offspring, possibly due to the limited exposure to a high-fat diet of only three weeks of fetal life relative to the 3-month period of maintenance on a low-fat diet (control diet). The lack of an effect of gestational high-fat programming was however surprising. Long-term low-fat diet feeding in male Wistar rats closely mimics the changes in normal middle-aged humans with no onset of disease [21]. HFG progeny closely mimicked the control progeny with similar metabolic and islet profiles demonstrating that a low-fat diet throughout postnatal life was sufficient to correct from adverse programming effects on islet morphology at earlier life stages. Hyperglycemia will diminish beta-cell integrity. The absence of overt hyperglycemia likely contributed to intact islet morphology in HFG progeny. Gestational high-fat programming may therefore be reversible, albeit temporarily, if a low-fat diet intervention is maintained but it is likely that metabolic and islet derangements will recur as these offspring age.

Gender influences biological outcomes [22] due to differences in the physiological systems of males and females [15]. The current findings indicated that male offspring were more susceptible to disease (evident by more adverse changes) after postnatal high-fat programming or gestational and postnatal high-fat maintenance compared to female offspring. For example, HFP females showed no significant changes in beta-cell morphology in contrast to their male counterparts. Estrogen may protect females from metabolic insults which may explain why male offspring were more susceptible to metabolic changes. In a recent study, male mice were more susceptible to obesity and impaired glucose tolerance due to increased abdominal adiposity secondary to adipocyte hypertrophy whereas, in contrast, estrogen protected female mice by modulating genes regulating lipogenesis, lipolysis, and adipogenesis [23]. 

The present study has several limitations. We lacked data on islet morphology for HFGP females which limited our investigation on gender-specific effects. Another constraint was not investigating the sole effect of lactational high-fat programming. A major shortcoming was the low sample sizes in some groups. Some sample sizes were on the threshold for statistical analysis (*n* = 3) which increased variability and limited statistical power. Low sample sizes in some of the groups may have limited the finding of significant differences and resulted in type 1 errors. To address this caveat in future studies, we will increase dam numbers thus generating more offspring and allowing one pup per gender to be randomly selected from each dam. Higher sample sizes will reduce biological variability and enhance precision. The differences in sample sizes within groups were attributed to leptin and insulin assay sensitivity and to some sample degradation. The main focus of the study was however on islet morphology and can be further expanded by assessing beta-cell function in future experiments and identifying the proliferating islet cell type(s). 

## 5. Conclusions

Male offspring maintained on a postnatal high-fat diet presented a diabetogenic and mild obese phenotype. Male offspring maintained on a high-fat diet throughout gestation and postnatal life presented a severe obese phenotype. In contrast, with gestational high-fat programming, there was an absence of a diabetogenic and/or obese phenotype in both male and female offspring. These findings emphasize the importance of gestational and postnatal nutrition in shaping offspring health and disease outcomes and confirm the durable effects of high-fat programming.

## Figures and Tables

**Table 1:** Weight, fasting circulating leptin, glucose and insulin concentrations, and HOMA in 3-month-old male and female rats.

	Control	HFG	HFP	HFGP
Body weight (g)				
Males	235 ± 6.48 *n* = 36	279 ± 16.19 *n* = 6	301 ± 18.67* *n* = 13	402 ± 13.92^∗†‡^ *n* = 8
Females	200 ± 8.81 *n* = 8	215 ± 32.79 *n* = 3	263 ± 22.98 *n* = 7	—
Leptin (ng/mL)				
Males	0.65 ± 0.10 *n* = 8	0.50 ± 0.00 *n* = 3	4.94 ± 1.23* *n* = 7	12.20 ± 1.74^∗†‡ ^ *n* = 3
Females	0.65 ± 0.10 *n* = 8	0.60 ± 0.10 *n* = 3	4.80 ± 1.29* *n* = 7	—
Glucose (mmol/L)				
Males	7.3 ± 0.25 *n* = 40	9.7 ± 1.08 *n* = 10	11.2 ± 1.09* *n* = 17	10.8 ± 1.59* *n* = 8
Females	5.8 ± 1.81 *n* = 6	7.9 ± 1.61 *n* = 3	15.9 ± 0.67^∗†^ *n* = 7	—
Insulin (*μ*U/mL)				
Males	185.0 ± 41.36 *n* = 26	109.5 ± 30.47 *n* = 5	407.4 ± 65.01 *n* = 13	669.1 ± 420.10^∗†^ *n* = 3
Females	174.8 ± 79.39 *n* = 8	75.1 ± 5.19 *n* = 3	328.2 ± 24.67 *n* = 7	—
HOMA-insulin resistance				
Males	79.4 ± 19.53 *n* = 18	37.9 ± 9.31 *n* = 4	188.8 ± 30.47 *n* = 11	378.0 ± 181.10^∗†^ *n* = 3
Females	17.1 ± 2.02 *n* = 4	26.6 ± 6.97 *n* = 3	236.5 ± 52.87^∗†^ *n* = 3	—
HOMA-beta-cell function				
Males	1127 ± 317.4 *n* = 18	267 ± 55.0 *n* = 4	1753 ± 548.4 *n* = 10	1600 ± 1256.0 *n* = 3
Females	69 ± 20.3 *n* = 4	23 ± 7.4 *n* = 3	20 ± 2.1 *n* = 3	—

HFG: high-fat diet during fetal life; HFP: high-fat diet during postnatal life; HFGP: high-fat diet during both fetal and postnatal life.  Data are means ± SEM.  *P < 0.05 versus control; ^†^ *P* < 0.05 versus HFG; ^‡^ *P* < 0.05 versus HFP.

**Table 2:** Islet cell ratios, islet cell, and acinar cell proliferation in 3-month-old male and female rats.

	Control	HFG	HFP	HFGP
Beta-cell to alpha-cell ratio				
Males	2.71 ± 0.42 *n* = 3	2.70 ± 0.78 *n* = 3	3.08 ± 0.20 *n* = 4	5.07 ± 1.95 *n* = 3
Females	3.94 ± 0.64 *n* = 5	4.60 ± 0.54 *n* = 3	3.36 ± 0.51 *n* = 3	—
Alpha-cell to beta-cell ratio				
Males	0.39 ± 0.07 *n* = 3	0.48 ± 0.19 *n* = 3	0.33 ± 0.02 *n* = 4	0.26 ± 0.08 *n* = 3
Females	0.28 ± 0.04 *n* = 5	0.22 ± 0.02 *n* = 3	0.31 ± 0.04 *n* = 3	—
Islet cell proliferation				
Males	0.0023 ± 0.0003 *n* = 3	0.0065 ± 0.0005 *n* = 3	0.0125 ± 0.0039 *n* = 4	0.0087 ± 0.0005 *n* = 3
Acinar cell proliferation				
Males	0.0008 ± 0.00012 *n* = 3	0.0017 ± 0.0004 *n* = 3	0.1393 ± 0.0387^∗†§^ *n* = 4	0.0062 ± 0.0014 *n* = 3

HFG: high-fat diet during fetal life; HFP: high-fat diet during postnatal life; HFGP: high-fat diet during both fetal and postnatal life.  Data are means ± SEM.  *P < 0.05 versus control; ^†^ *P* < 0.05 versus HFG; ^§^ *P* < 0.05 versus HFGP.
